# S8-2 Health Promoting Sports Federations: theoretical foundations and guidelines

**DOI:** 10.1093/eurpub/ckad133.039

**Published:** 2023-09-11

**Authors:** Aurélie Van Hoye, Susanna Geidne, Anne Vuillemin, Kieran Dowd, Iva Glibo, Sandra Heck, Bjarne Ibsen, Stacey Johnson, Melanie Kingsland, Sami Kokko, Aoife Lane, Linda Ooms, Marie Overbye, Catherine Woods, Geraldine Zeimers, Stephen Whiting, Mathieu Winand

**Affiliations:** University of Limerick, Ireland; Örebro University; Université Côte d'Azur; Technological University of the Shannon; ENGSO Youth; University of Luxembourg; Southern Denmark University; Institut de Cancérologie de l’Ouest; University of Newcastle; University of Jyvaskyla; Technological University of the Shannon; Mullier Instituut; University of Stirling; University of Limerick; Université Catholique de Louvain; World Health Organisation; Lunex University

## Abstract

**Purpose:**

The potential for organised sports to promote health has been underexploited so far, according to researchers and policy-makers. Sports clubs have limited capacity to promote health due to their voluntary nature and have called for support from their national sports federations. The present article provides guidelines based on health promoting sports clubs theoretical foundations, and an analysis of practical tools and proven strategies to support national sports federations’ investment in health promotion (HP).

**Methods:**

A qualitative iterative study was undertaken, based on five two-hour meetings of a group of 15 international researchers in health promotion in sports clubs. Notes and minutes from meetings, as well as shared outputs were analyzed based on the health promoting sports club framework.

**Results:**

Guidelines for national sports federations to promote health includes a definition of a health promoting sports federation, a description of the settings-based approach stages adapted to national sports federations, as well as practical applications of the health promoting sports club’s intervention strategies. The analysis of existing tools demonstrated that most tools are centered on a single dimension of health (social, mental, physical, spiritual or community), and often on a specific health topic. Furthermore, they do not cover HP as a continuous process or support of sports clubs’ members health, but are generally short-term programs. The HPSF clarifies theoretical concepts, their practical implementation via case studies and outlines intervention components and tools useful for sports federations in their implementation of HP.

**Conclusions:**

The guidelines are meant to facilitate national sports federations to acknowledge, reinforce and foster their further investment in HP.

**Funding source:**

This project has received funding from the European Union’s Horizon 2020 research and innovation programme under the Marie Skłodowska-Curie grant agreement No 101028401. This work was funded by a grant from the World Health Organization in partnership with Santé publique France, Université de Lorraine and Université Côte d’Azur.

